# Correspondence: Reply to ‘SEMA4A variation and risk of colorectal cancer'

**DOI:** 10.1038/ncomms10695

**Published:** 2016-03-10

**Authors:** Heinz Sill, Eduard Schulz, Verena Steinke-Lange, C. Richard Boland

**Affiliations:** 1 Division of Hematology, Department of Internal Medicine, Medical University of Graz, Graz A-8036, Austria; 2 MGZ—Center for Medical Genetics, D-80335 Munich, Germany; 3 Division of Gastroenterology, Baylor University Medical Center, Dallas, Texas 75246-2017, USA

Kinnersley *et al*. comment on our recently published study^1^ and report a failure to support semaphorin A (*SEMA4A*) variations as risk alleles for colorectal cancer (CRC)^2^.

The authors investigated the contribution of the recurrent *SEMA4A* variants p.Gly484Ala (rs148744804) and p.Pro682Ser (rs76381440) in cohorts of CRC cases from different European populations. No significant association could be found in any of their series. In our study, we have focused on the p.Pro682Ser single-nucleotide polymorphism exclusively in patients with familial CRC type X (FCCTX) and used controls with absent personal history of cancer. FCCTX is a distinct disease entity characterized by families meeting Amsterdam criteria and CRCs lacking mismatch repair defects. The genetic background of FCCTX is regarded heterogeneous^3^,^4^. We have, indeed, taken the pitfalls of population stratification into account and confined our analysis to FCCTX patients from Germany and Austria. To validate our finding of a significant association between p.Pro682Ser and risk of FCCTX, data on FCCTX patients from the German cohort investigated by Kinnersley *et al*. might be added.

Protein-changing variants in *SEMA4A* were found at comparable frequencies in both, controls and a familial early-onset CRC cohort that included FCCTX patients. It is well perceived that not all variants of a tumour-associated gene are pathogenic, which has also been shown for *POLD1* recently^5^. We used the prediction tools PolyPhen-2, SIFT and PROVEAN to classify variants of their study into benign and deleterious. When taking variants classified as deleterious by all three algorithms (Ile101Thr, Lys219Asn, Arg267Lys, Met434Thr, Phe482Ile and Tyr680His) and frameshift mutations together, they were statistically significantly more frequent in cases than controls (11 of 1,006 cases versus 5 of 1,606 controls, *P*=0.0183, Fisher's exact test, two tailed).

With regard to the *SEMA4A* p.Val78Met identified by our group in a FCCTX kindred comprising 88 family members, functional *in vitro* data corroborated its pathogenicity. In contrast to developmental disorders, incomplete penetrance of a putative disease susceptibility variant is frequently observed in familial cancer syndromes. Nevertheless, the fact that protein-changing variants, including frame shifts, occur in controls deserves further attention. Here clinical data on the particular individuals as well as his/her families would be helpful. Although patients with FCCTX show a median age of >60 years at disease onset^4^, which is higher than that of individuals of the ‘1958 Birth Cohort', it might well be that some of them have already developed tumours or represent yet unaffected members of cancer prone families.

In summary, we appreciate the valuable contribution of Kinnersley *et al*. on *SEMA4A* variants in sporadic and familial CRCs. In the FCCTX syndrome, however, they remain in our view susceptibility candidates that are encountered rarely or even constitute ‘private' variants to particular families, as has been described^6^,^7^. We agree that inclusion of *SEMA4A* in clinical screening and surveillance programs is not justified at present.

## Additional information

**How to cite this article:** Sill, H. *et al*. Correspondence: Reply to ‘SEMA4A variation and risk of colorectal cancer'. *Nat. Commun*. 7:10695 doi: 10.1038/ncomms10695 (2016).
